# Sequential Subterranean Transport of Excavated Sand and Foraged Seeds in Nests of the Harvester Ant, *Pogonomyrmex badius*

**DOI:** 10.1371/journal.pone.0139922

**Published:** 2015-10-28

**Authors:** Walter R. Tschinkel, William J. Rink, Christina L. Kwapich

**Affiliations:** 1 Department of Biological Science, Florida State University, Tallahassee, Florida, United States of America; 2 School of Geography and Earth Sciences, McMaster University, Hamilton, Ontario, Canada; Arizona State University, UNITED STATES

## Abstract

During their approximately annual nest relocations, Florida harvester ants (*Pogonomyrmex badius*) excavate large and architecturally-distinct subterranean nests. Aspects of this process were studied by planting a harvester ant colony in the field in a soil column composed of layers of 12 different colors of sand. Quantifying the colors of excavated sand dumped on the surface by the ants revealed the progress of nest deepening to 2 m and enlargement to 8 L in volume. Most of the excavation was completed within about 2 weeks, but the nest was doubled in volume after a winter lull. After 7 months, we excavated the nest and mapped its structure, revealing colored sand deposited in non-host colored layers, especially in the upper 30 to 40 cm of the nest. In all, about 2.5% of the excavated sediment was deposited below ground, a fact of importance to sediment dating by optically-stimulated luminescence (OSL). Upward transport of excavated sand is carried out in stages, probably by different groups of ants, through deposition, re-transport, incorporation into the nest walls and floors and remobilization from these. This results in considerable mixing of sand from different depths, as indicated in the multiple sand colors even within single sand pellets brought to the surface. Just as sand is transported upward by stages, incoming seeds are transported downward to seed chambers. Foragers collect seeds and deposit them only in the topmost nest chambers from which a separate group of workers rapidly transports them downward in increments detectable as a "wave" of seeds that eventually ends in the seed chambers, 20 to 80 cm below the surface. The upward and downward transport is an example of task-partitioning in a series-parallel organization of work carried out by a highly redundant work force in which each worker usually completes only part of a multi-step process.

## Introduction

Ants are among the most abundant and important animals that burrow in the world's soils, moving huge amounts of soil in the process of excavating their nests. As a result, they are the chief agents of bioturbation and biomantling in many temperate and tropical ecosystems [1–3]. Depending on species and colony size, nests may range from small and shallow [4, 5] to colossal and deep [6, 7]. The architecture of these subterranean nests is species-typical, usually consisting of modifications of horizontal chambers connected by more-or-less vertical shafts [4, 5, 8–10]. Because these nests are constructed in the dark by groups of workers that lack a plan and a leader, there has been much interest in how nest excavation and/or relocation is self-organized, and the process has been studied in the laboratory [11–16].

The hallmark of social insects is division of labor, in which different groups of workers are specialized to perform *different* tasks. However, there are also many examples of task partitioning, in which different workers carry out parts of the *same* task. Examples include sequential transport of leaf pieces by leaf-cutter ants (*Atta* spp.), foraging, management of waste, soil transport during nest excavation, and trail construction [16–22]. The benefit of such partitioning may include increased speed of completion and increased efficiency, especially if the sequential transfer is made non-randomly [23]. The cost of partitioning may include efficiency compromises [24]. When applied to waste, task partitioning may improve colony hygiene by reducing the likelihood of pathogen transfer into the nest [25–27]. Partitioned tasks tend be those that are more complex [28], and whose subtasks can be completed in sequence.

The study of nest architecture intersects with archeology, soil science and geology because nest construction moves sediment from depth to the surface, or deposits it below the surface, thus rearranging the sediment layers in unpredictable ways. This rearrangement is particularly important to a sediment dating method called optically stimulated luminescence (OSL). Quartz grains buried in the dark are exposed to radiation from sources as varied as cosmic rays to various radioactive elements. This radiation causes electrons to be trapped in crystal defects in proportion to exposure, so that the longer a grain has been buried, the more electrons it accumulates in traps. If grains are exposed to light in nature during deposition, or when transported by ants to the surface, they lose the electrons previously accumulated in traps (natural zeroing). Analogously, when exposed to a light in the laboratory, previously stored electrons emit their energy as UV light, and the grain gives up its UV-emitting capacity (laboratory zeroing). Absent sediment disturbance, the deeper a grain is buried, the longer it has been irradiated and the more UV it can emit, thus creating a method for dating sediment layers in the absence of datable carbon [29, 30]. The amount of UV light emitted can be converted to a previous natural radiation dose using controlled laboratory irradiation. The OSL age of the sediment is determined by the ratio of laboratory radiation dose to natural radiation dose rate, and can be used to establish an age estimate for the burial of grains since last light exposure. These age estimates can be supplemented with any other datable materials (biological, geological or archaeological) deposited within an undisturbed sequence.

When burrowing animals bring sediment from deeper layers to the surface, they fuel the process of biomantling, or sediment turnover, a process important in mineral redistribution and sediment mixing. Ants are among the most important biomantling agents in the warmer regions of the world (see [1] for a review) replaced by earthworms in cool temperate regions [31, 32]. Rates of biomantling by ants have been estimated for several of the world's sediments (reviewed in [1]). Historically, ant colonies have been considered more-or-less sedentary, but since the 1980s, it has become increasingly apparent that many (if not most) species move fairly often [33, 34]. Tschinkel [35] quantified the rate and process of nest relocation in the Florida harvester ant (*Pogonomyrmex badius*) in the sandy sediments of the Florida coastal plains, and estimated the resulting biomantling rate for a population of about 400 colonies. Colonies moved an average of 4 m once a year, creating a new nest up to more than 2 m deep, and moving the entire colony into it in a period of 4 to 6 days. Nevertheless, in spite of its much smaller worker and colony size, the co-occurring fungus gardener, *Trachymyrmex septentrionalis* [35, 36] moves many times as much sediment to the surface as the harvester ant, but the harvester ant brings up much deeper sediments, bringing attention to the fact that the effects of biomantling must be understood both in terms of amount and depth distribution of the biomantled sediments.

Of much greater importance to the method of OSL than biomantling is subterranean transport of sediment from one layer to another without exposure to light (and therefore without zeroing) [37–41]. An experiment in which a harvester ant colony was planted in sediment consisting of 12 different colors in layers down to a depth of 2 m showed that a significant amount of sediment from deeper layers was deposited in shallower layers in the dark, and lesser amounts from shallower to deeper layers [42]. If significant biomantling along with upward subterranean transport has occurred, the OSL age of the sample can be estimated from the ages of the youngest grains sampled (using the OSL minimum age model [30]). This can be compromised if there has also been a significant amount of downward transport from the surface into the sample, which can cause the OSL age based on the minimum age model to be even younger than the true burial age.

The method of Rink et al. [42], that is, of planting a harvester ant colony in a "layer cake" of different colors of sand, can illuminate not only the processes affecting OSL dating, but also several aspects of the process of nest excavation by the ants. This paper repeats the experiment of Rink et al. [42] with careful quantification of surface and below-ground sand deposition, as well as analysis of the progress of the excavation, the mixing of sediment in the process, the mode of upward sediment transportation by workers, the related process of downward seed transportation, and the overlap of sand removal and seed deposition behavior by workers of the Florida harvester ant.

## Materials and Methods

### Study site

The study population of Florida harvester ant, *P*. *badius*, is located in a 23 ha site (latitude 30.3587, longitude -84.4177) about 16 km southwest of Tallahassee, Florida, USA, within the sandhills ecotype of the Apalachicola National Forest. The site, Ant Heaven, consists of excessively drained sandy sediment occupying a slope to a wetland and stream, causing its water table to be depressed (>5 m at the maximum), thereby making it suitable for *P*. *badius* and *Solenopsis geminata*, as well as several drought-resistant species of plants such as *Opuntia* and *Nolina*. The forest consists of longleaf pines (*Pinus palustris*) planted ca. 1975, turkey oak (*Quercus laevis*), bluejack oak (*Quercus incana*), occasional sand pines (*Pinus clausa*) and sand live oak (*Quercus geminata*). Because the sediment had been disturbed in the early 1970s, the natural ground cover of wiregrass (*Aristada stricta*) was absent, replaced by broomsedge (*Andropogon* spp.) and several other successional species of grasses, herbs and shrubs. The same disturbance may have helped establish this dense population of *P*. *badius*, whose nests are easily spotted because the ants decorate the excavated sediment disc with a layer of charcoal bits (mostly the ends of burned pine needles). The black charcoal contrasts sharply with the light-colored sand or litter.

This project was carried out under US Forest Service, Apalachicola National Forest permit number APA56302, Expiration Date: 12/31/2017. *Pogonomyrmex badius* is not a protected species.

### Colored sand "layer cake" experiments

**Fig 1:**
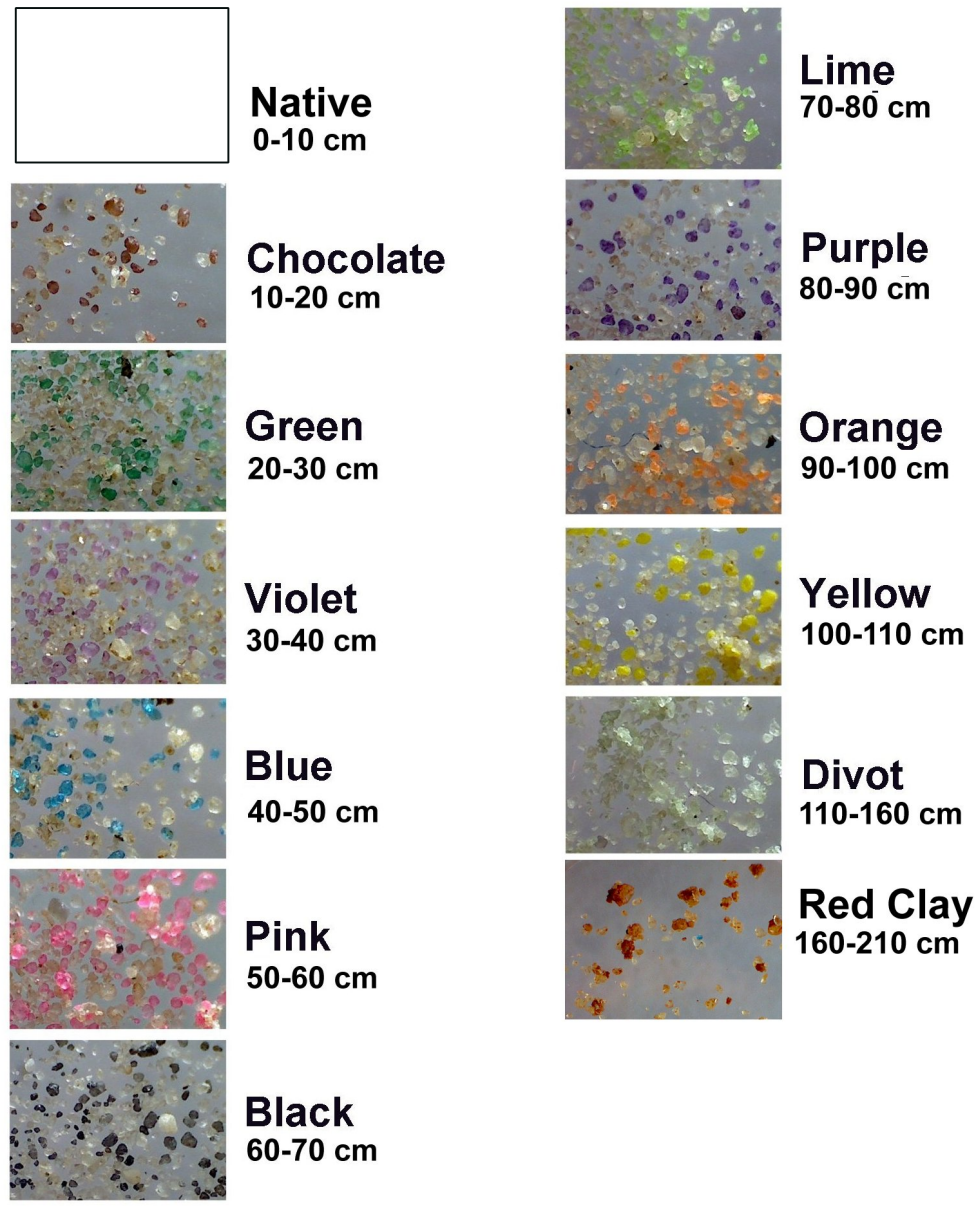
The sand colors, their vertical order and depth. Each layer consisted of 25% colored sand mixed with 75% native sand. The bottom two layers were each 50 cm thick, and all layers above these were 10 cm thick.

In November 2010, a *P*. *badius* colony at Ant Heaven was induced to excavate a nest through multiple layers of colored sand, and was excavated in May 2011. These results were published by Rink et al. [42]. This experiment was replicated with a second colony in 2011. A 2 m deep pit measuring 1 m by 2 m in horizontal aspect was excavated, one half of which (1 m x 1 m x 2 m) was lined on 3 sides with plywood. Within this plywood box, beginning at 2 m depth, 12 layers of sand, each a different color, were laid down, flattened and tamped. The first two layers were each 50 cm thick, but all layers shallower than 1.1 m were 10 cm thick. The open side of each layer was bounded by a plank, and the work pit filled with native sand to that level before the next colored layer was installed. The colored sand was purchased from Sandblast Entertainment in Pensacola, Florida, and was mixed with 3 parts native sand from the pit (i.e. final composition, 25% colored sand) before emplacement in the pit. The sand colors and the order and depth of their layers is shown in Fig 1.

**Fig 2:**
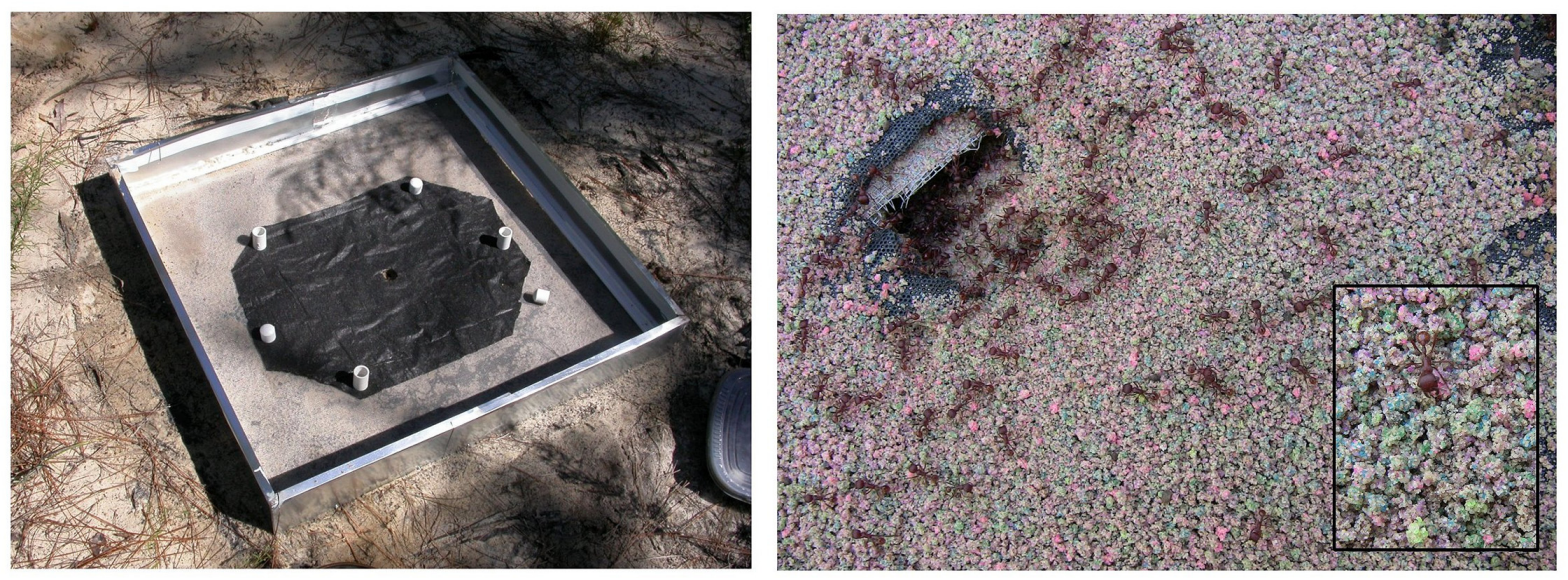
A. The screen-bottom box into which the *P*. *badius* colony was released to dig a nest through a central hole in the screen into the "layer cake" below. The black weed matte allowed the easy collection of the sand the workers brought to the surface every day. B. An example of the sand brought up to the surface by the ants during one day. The inset shows a detail of colored sand pellets dropped by the ants.

A colony of *P*. *badius* (10,000 workers, no brood) was excavated from nearby in Ant Heaven and planted in the "layer cake" on October 16, 2011. A 1 m by 1 m x 20 cm screen-bottom galvanized metal box (Fig 2A) was placed on top of the layer cake, and in a rough simulation of a natural nest relocation, about 1000–2000 ants from the excavated colony were released into it daily for 5 days until the entire colony had been installed. The queen was added in the last batch. The screen had a hole in the middle through which the ants gained access to the sand beneath, and began immediately excavating a nest, induced through a few "starter holes" made with a stick.

A layer of weed mat with a central hole exposing the nest opening was placed on the screen bottom so that any sand dumped by the ants could be collected easily (Fig 2B). Initially, sand excavated by the ants was collected daily, and later at longer intervals. The weights of the sand were used to estimate the rate and progress of excavation on a daily basis. Dividing the sand weight by 1.5 (the bulk density of dry sand) resulted in the daily volume of chambers created, and summing these over time gave the cumulative total nest volume.

Each collection of sand was dried and weighed, and a subsample was taken for later determination of the frequency of each color of sand. In addition, from Oct. 21 to Nov. 13, 2011 approximately 50 randomly selected intact sand pellets were collected daily and placed into the wells of well plates for later determination of their color composition and grain count (see below).

#### Excavation of the Layer Cake Colony

**Fig 3:**
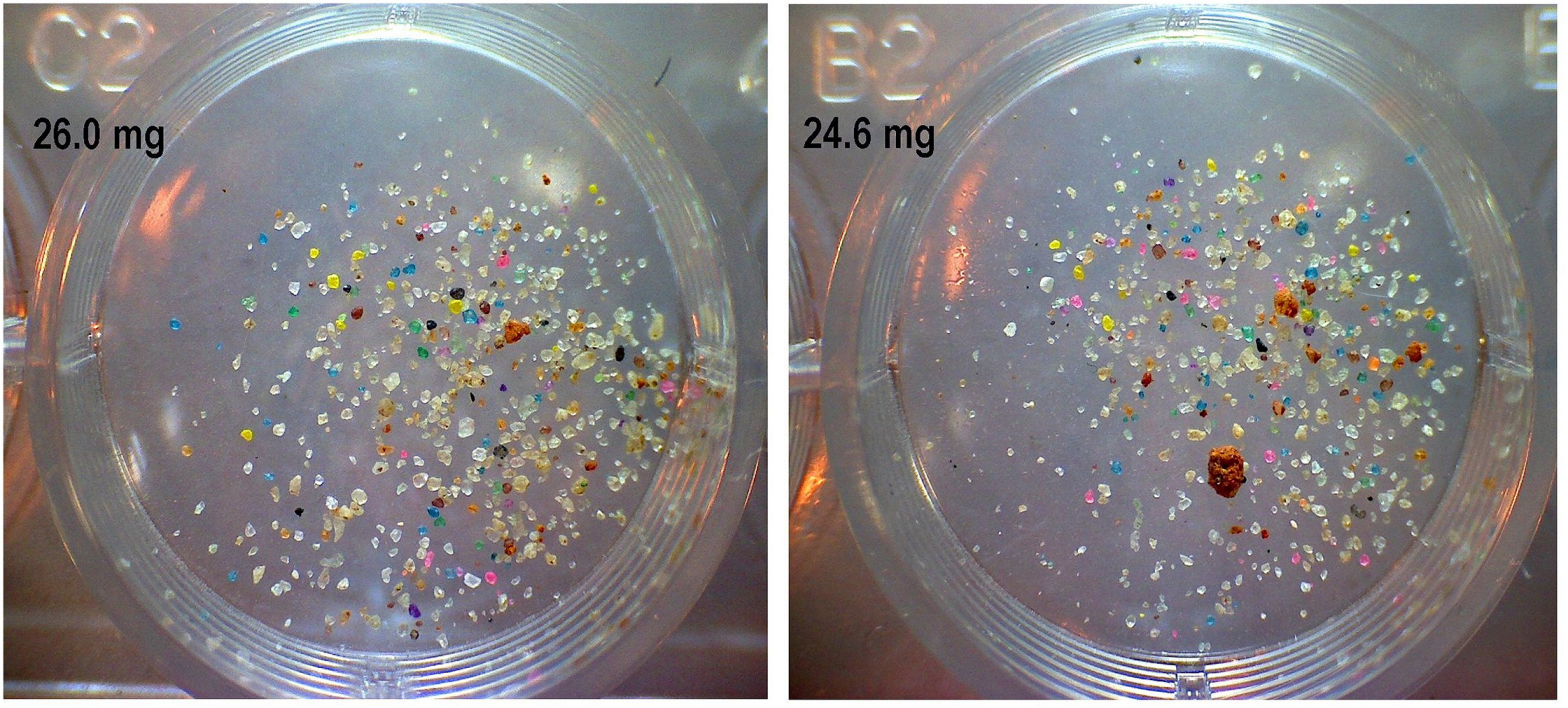
Examples of two small samples of daily sand collections placed into wells in well-plates. The weight of each sample is indicated. Colored and native grains were counted in such samples to determine the rate and location of digging in the colored layers.

In May 2012, the layer cake colony was excavated chamber by chamber by digging a pit next to it and lifting off horizontal layers of sand with a brick trowel, exposing each chamber in turn. All chamber contents were collected, and the chamber outline traced on a sheet of transparent acetate. Any non-host sand (that is, below-ground deposition) was photographed, then collected, dried, weighed and the colored grains counted as follows: twelve small samples (15–25 mg) were scattered in the bottoms of well-plates and photographed (Fig 3). The number of colored and native grains in each sample was counted, and from the proportion of each color, the weight of each color in the sample was calculated. Applying the counts-to-weight conversion and multiplying by 4 (only one-fourth of the sand was colored, 3/4th was native) produced the weight of non-host sand (and its color) deposited in each chamber or shaft.

#### Color analysis of daily samples

The dried daily samples were carefully mixed, random samples of 15–25 mg were weighed on a Cahn Electrobalance and placed into well plates. Each well containing one sample was then photographed with an Emcal digital microscope (EmCal Scientific, Inc., San Diego, CA 92198), and the number of grains of each color (plus native grains) was counted in the images (Fig 3). These counts provided a record of the progress of nest excavation. The first appearance of colors revealed the maximum nest depth on that day, and the proportion of the colors revealed the relative rates and location of excavation in the different layers. Cumulative sand weights revealed the total chamber volume and the depth for each day.

#### Color analysis of sand pellets

Each individual sand pellet was placed into a separate well in the well-plates and photographed with a digital microscope and counts of the number and color of sand grains made from the images, as above. Because each sand layer was homogeneous in color, individual sand pellets should contain mostly a single color of sand if the newly-formed pellets are carried directly to the surface. Therefore, mixing of colors in the pellets would suggest that sand was deposited and reformed into pellets on the way to the surface.

The degree of color mixing of each pellet was estimated by a count of the number of different colors, and by calculating a Gini coefficient as follows. The number of sand grains of each color was converted to a percentage of the total colored grains (native grains were excluded). The percentages were ranked in ascending order and cumulated so that color by color, the percent of colored grains increased from 0 to 100%. These cumulative curves were compared to a theoretical curve of complete evenness in which every color was represented by the same percentage, i.e. formed a straight line between 0 and 100% as colors were cumulated. The differences between the observed cumulative curve and this curve of complete evenness produced the Gini coefficient. Complete evenness resulted in a curve identical to the theoretical and a coefficient of zero, while a single color resulted in a coefficient of 1.0.

#### Sand properties

Bulk properties of the sand mixtures were determined by volume and weight after moderately packing the sand into tubes. Quartz density is approximately 2.65 g/cm^3^, and dry packed sand had a density of 1.5, suggesting about 40% pore space. Packed sand columns were then saturated with water, allowed to drain and reweighed to determine the water-holding capacity and pore space.

Combining grain counts of weighed samples with bulk density measurements linked the weight of excavated sand with the number of grains excavated. The area of each colored sand grain was measured and recorded using the program ImageJ (http://imagej.nih.gov/ij/). Grain volume was estimated by assuming that the third dimension was approximately the radius of a circle with the measured silhouette area. Volumes averaged about 0.015 mm^3^ (s.d. 0.013).

### Experimental tests of caching

#### Seed caching

Seven additional *P*. *badius* colonies at Ant Heaven (not the Layer Cake colony) were offered 100 to 250 native seeds that had been sprayed with a 10% suspension of fluorescent printers ink (Gans Co., http://www.gansink.com/locations.asp) in diethyl ether. These marks were not visible under normal light, but fluoresced brightly under UV. Each seed offering was marked with a different color of fluorescent ink (orange, green, blue, yellow). Depending on the replicate, colonies were allowed to collect and move each offering of seeds for two days, one day, and finally 15 min. to 1.5 hr before the colony was excavated. Ants readily collected the seeds despite the presence of ink. After the last seeds were offered, the nest was carefully excavated chamber by chamber [8, 35], collecting the seeds in each. All seeds, including those in the seed storage chambers were checked for marks under UV light.

#### Sand caching

A pit was dug adjacent to each of several colonies at Ant Heaven (not the Layer Cake colony). At a depth of about 30–50 cm, sand was excavated laterally until a chamber was (barely) intersected. Damp pink fluorescent sand was then pushed into this chamber, and the pit filled in again. One or two days later, the appearance of pellets of pink sand on the ground surface indicated that the colony was re-excavating the partly-filled chamber. After 2 days, each colony was carefully excavated chamber by chamber, the chamber outlines and placement of pink sand grains were traced on transparent acetate and photographed.

### Overlap in seed and sand transport

For two additional colonies, forager and sand worker population sizes were estimated using the Lincoln index mark-release-recapture method [43]. Foragers were identified as individuals that collected seed or cookie bait 150 or more cm from their nest mound before making a return trip, while sand transporters were identified as individuals that emerged from the nest entrance and deposited a bolus of sand on the nest surface. To determine if sand removal and foraging were performed by separate labor groups, foragers and sand-transporters were marked separately over two, successive 48 hour periods using different colors of fluorescent printer’s ink (for more detailed methods, see [44, 45]). The forager population was sampled again after twenty days to determine if sand-worker’s tendency to forage increased with age.

## Results

**Fig 4:**
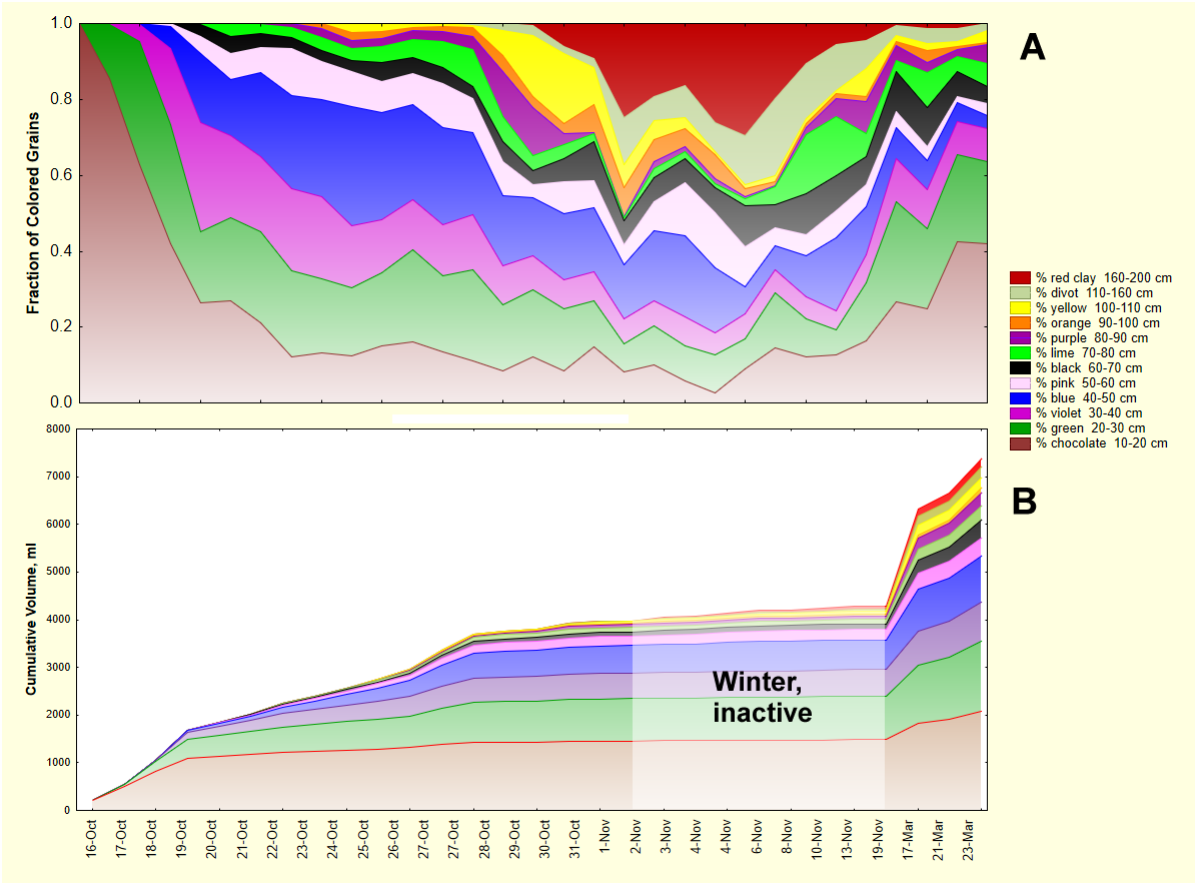
The progress of nest excavation. A. The proportion of each color of sand in the daily samples collected from Oct. 16, 2011 to the end of March 2012. The first appearance of each color indicates the maximum depth that the nest had penetrated, and the proportion of each color relates the relative rate of excavation in each colored layer. B. The daily sand weights in Fig 4A were converted to volumes and cumulated over the entire period, yielding the relative volume of chambers in the various layers. Only small amounts of sand were deposited between Nov. 1 and early March, a period during which the colony was largely inactive and broodless.

The planted *P*. *badius* colony immediately began excavating a nest through the screen bottom cage and into the multi-colored layer cake (Fig 2A and 2B). The changing color composition of the sand collected daily revealed the maximum depth to which the nest had penetrated and the relative chamber volume created in each color layer that day (Fig 4A and 4B). Within two days, the nest had penetrated to the bottom of layer 4 (violet, 30–40 cm) and by the third day, it had just touched the layer 7 (black, 50–60 cm). By 14 days, it had reached layer 13 at 150 cm (red clay) and beyond. A moving vertical section of Fig 4A reveals the relative rate of excavation in the colored layers. Initially, all excavation was in layer 2 (chocolate, 10–20 cm), but by the third day, this made up half or less of the excavated sediment. By 7 days, layers 2–5 (chocolate, green, violet and blue, respectively) made up more or less equal shares of the excavated sand, with layers 6–9 (pink, black, lime and purple, respectively) making up lesser amounts. At the end of 2011 (Nov. 13), all depths were being accessed, and brought to the surface, with no clear favorite except perhaps layer 2 (chocolate,10–20 cm). Once excavation began again in March 2012, the colors appeared in rough proportion to their depths, with layers 2–5 (chocolate, green, violet and blue, respectively) predominating in that order, with lesser amounts of layers 6, 8–12 (pink, lime, purple, orange, yellow and divot, respectively). The relatively large amount of layer 7 (black) may be partially an artifact caused by mistaking natural soil charcoal for black sand (see below). The rates of excavation in spring 2012 suggest evenly distributed renovation or enlargement of the nest in proportion to the existing chamber volume.

In Fig 4B the weight of excavated sand has been converted to cumulative volume of nest created. The autumn slow-down in nest enlargement is apparent, and by the time excavation ceased in Nov. 2011, the total nest volume was about 4 L. After the winter lull, this was enlarged to about 8 L. This is in line with the expected volume of a nest created by about 10,000 ants [8]. The relative and absolute chamber volume in each layer is also apparent in Fig 4B.

**Fig 5:**
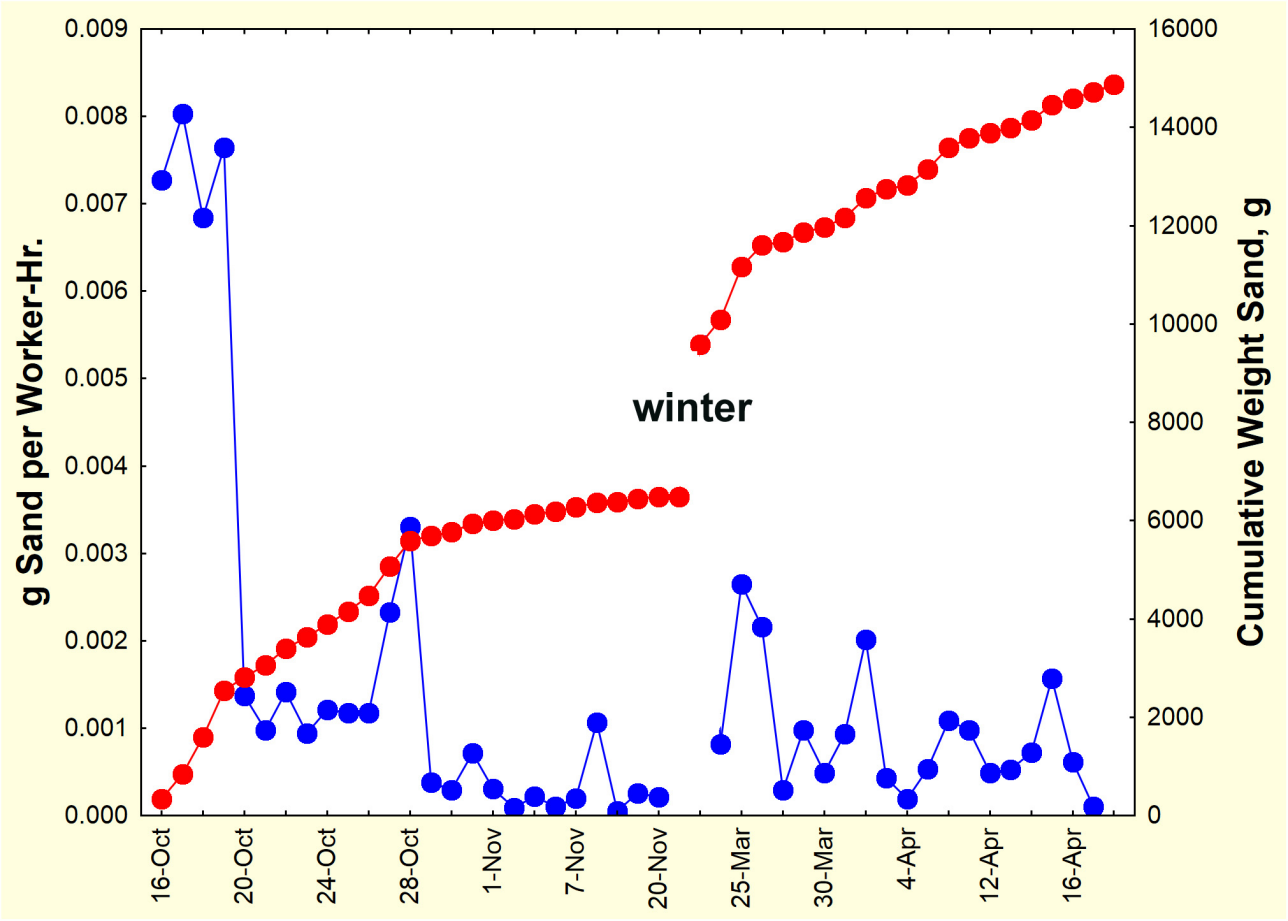
Sand deposition rate and cumulative deposition. The per-worker rate of digging and transport was initially very high, then varied from day to day. The cumulative weight of sand excavated increased rapidly for about two weeks, and then again after a winter lull.

The absolute rate of sand excavation can be seen in Fig 5. This rate was computed by dividing the weight of sand collected daily by the number of workers in the colony on that day (workers were added during the first 5 days, so that the number present was 2000, 3000, 4100, 5600, 7200 and 10,000). Soil damp enough for grain adhesion is carried in pellets, but ants in the genus *Pogonomyrmex* also possess a psammophore (basket of setae) on the ventral surface of the head, which allows them to transport more than two times as much dry sand per load than with mandibles alone [46]. Species of *Pogonomyrmex* are prolific excavators of soil. In this study excavation occurred at a rate of approximated 7.5 mg per worker per hr, but by the 4th day, this had dropped to about 1.5 mg per worker-hr, and continued to decline (with one excursion) until digging ceased in late November. It should be noted that these rates per worker were based on the entire worker population within the nest, so that the divisor increased as workers were added. It is unlikely that all these workers engaged in excavation. Rather, it is likely that a minority of workers ferried sand to the surface, and the weight of sand per actively ferrying workers was much higher.

**Fig 6:**
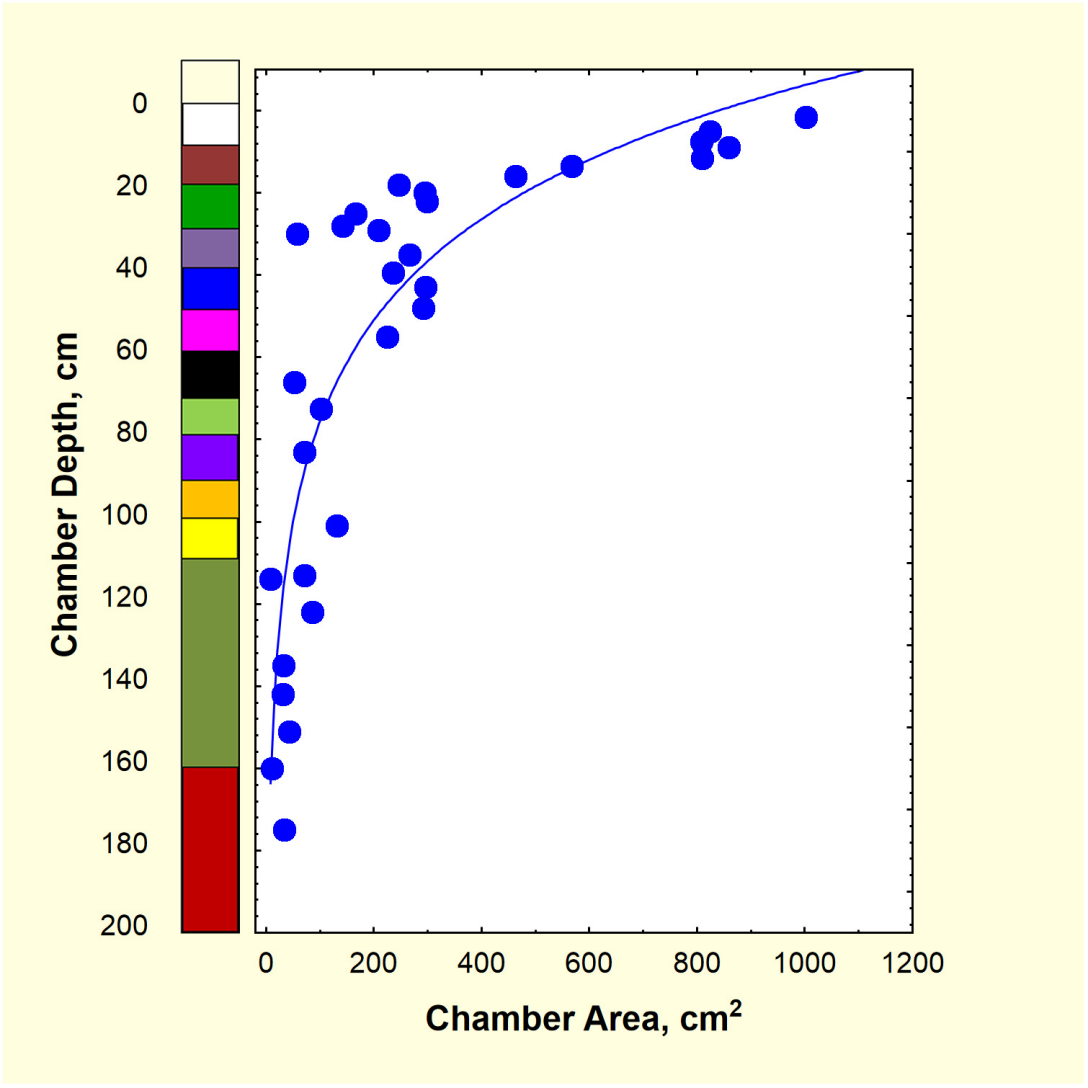
Chamber area in relation to chamber depth. The chamber area decreased logarithmically with chamber depth, giving the distribution of area its species-typical shape[8]. The colored layers are shown on the left axis.

Although the total size of the nest met expectations, the details of the architecture deviated from those of a normal nest constructed during nest relocation [35]. In particular, there were more shafts and small chambers than expected giving the nest a less crisply organized character than usual. However, the distribution of total chamber area in relation to depth was similar to natural nests, with chambers decreasing in size and number with depth in a quasi-logarithmic fashion (Fig 6). Fig 6 also shows the location of chamber area in relation to the colored layers. In spite of the deviation of architectural details, the distribution of chamber volume in relation to depth was normal, and therefore, the colors in Fig 3A and 3B reveal the process of natural nest excavation.

### Below-ground deposition of sand

Careful excavation, layer by layer, of the nest in May 2012 revealed that a substantial amount of colored sand was deposited in host layers of other colors, below ground (subterranean deposition). All non-host sand (i.e. sand of a color other than the layer in which it resided) was collected, identified as to origin, dried, weighed and each color of sand grain counted to estimate below-ground deposition. Because colored grains only made up 25% of each colored sand layer, these values were multiplied by 4 to derive the actual weight of each color in the sample.

**Fig 7:**
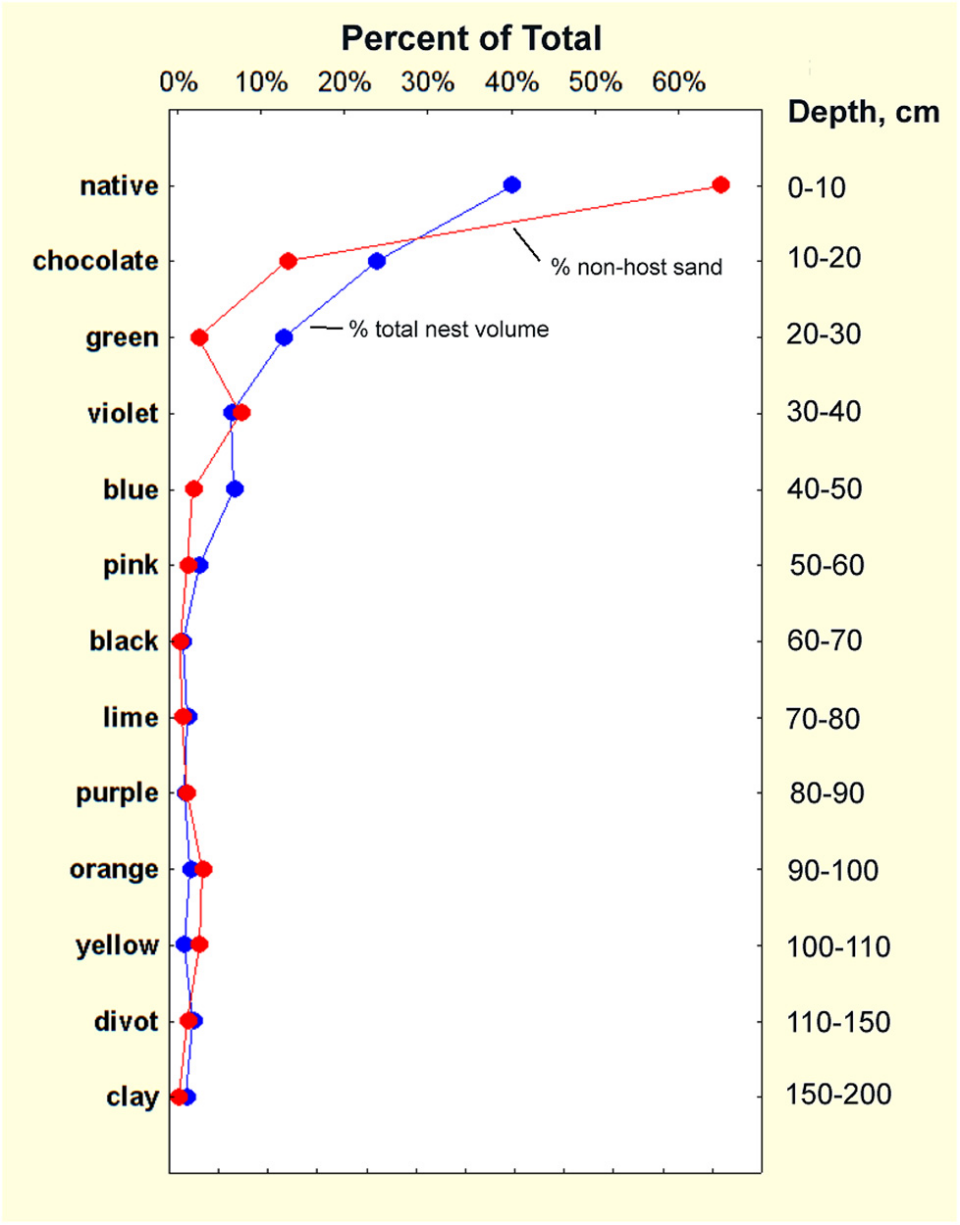
Fraction of total chamber area by depth, along with deposition of non-host sand. The chamber areas in Fig 6 were cumulated by decile (tenths of the maximum depth) and show the characteristic distribution of nest volume by depth (blue symbols and line). The distribution of the deposition of non-host sand (red symbols and line) is superimposed over the volume distribution. Of the 13 kg of sand excavated to form the nest, 2.5% was deposited in the dark below the surface.

**Fig 8:**
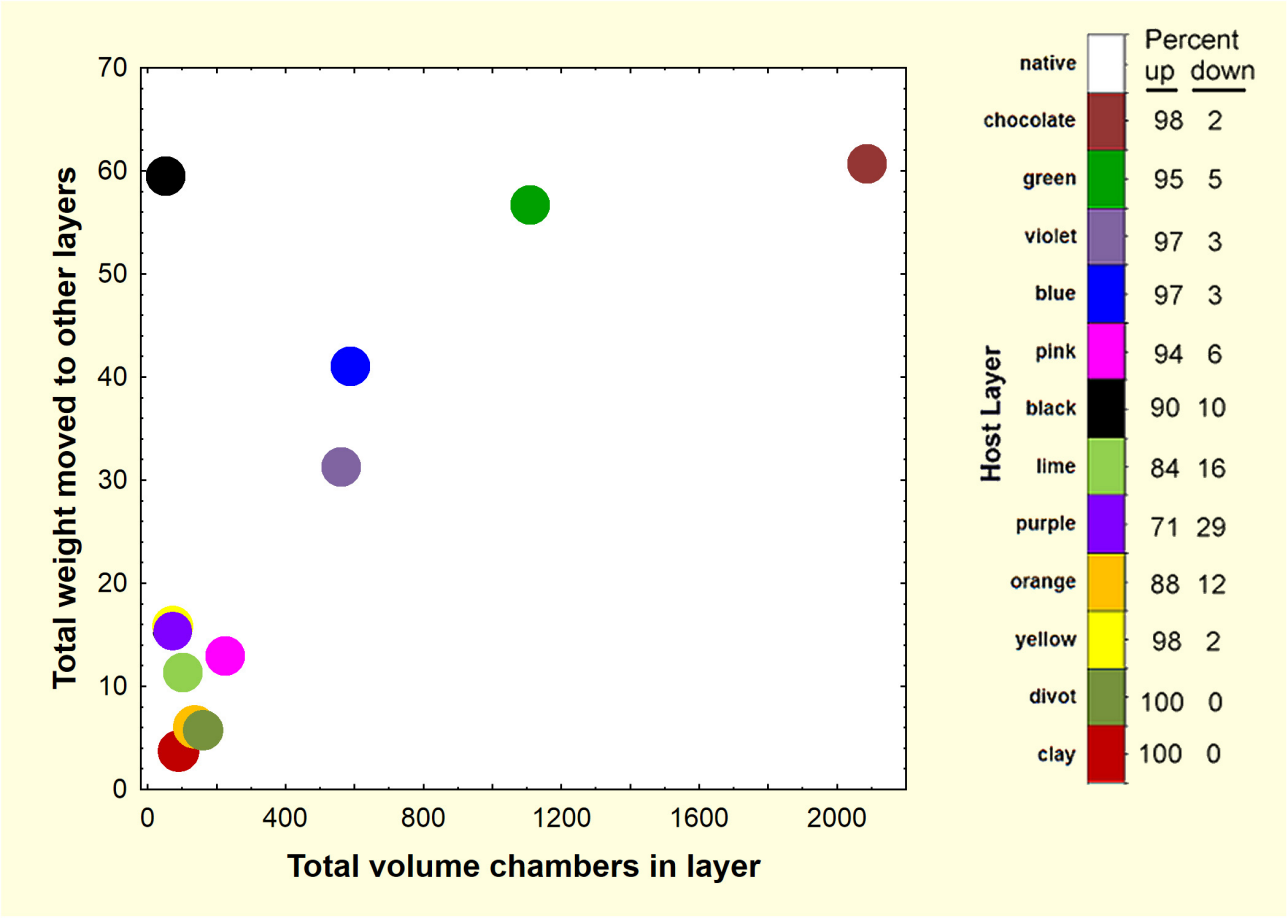
The total weight of below-ground deposition of the different sand colors was positively related to the volume of chambers excavated in that layer. In view of the small volume of chambers found in the black sand layer upon excavation, the large weight of the black sand was probably an error caused by mistaking natural charcoal for black sand. The vertical order of sand colors is shown on the right, as is the percent of the subsurface deposition moved up and down. Each point reflects that color in a non-host layer.

**Fig 9:**
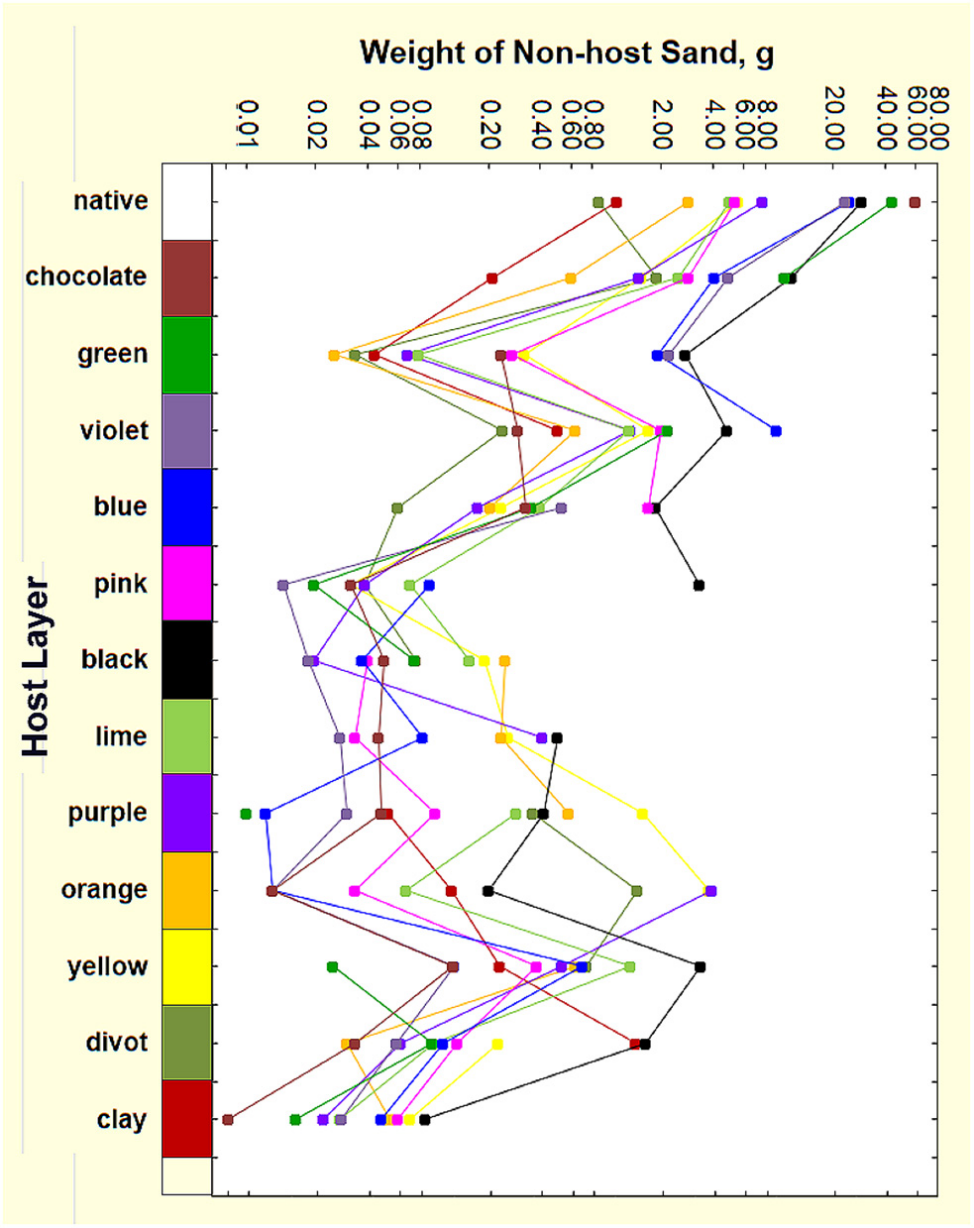
Below-ground deposition of colored sand in non-host layers. The colors of the lines correspond to the colored layer bar on the left axis. Note the log scale on the x-axis. Deposition is greatest in the top 30 cm of the nest, with a minimum in the pink to lime region.

Summing all of the non-host sand deposited below ground (321 g) showed that about 2.5% of the 13 kg of excavated sand was deposited below ground, approximately 75% of it in the upper 30 cm of the nest (Fig 7). If this deeper sand were mixed homogeneously in the top 30 cm of sediment (assuming the nest is within a cylinder 40 cm in diameter), then 4 of every 1000 sand grains would be alien to this layer. The source of this non-host sand was approximately proportional to the volume of chambers the ants had excavated in the source layer (Fig 8), with chocolate being proportionately less relocated. Thus, deposition diminished the greater the vertical separation from the source chambers below (Fig 9). For example, 60 g of layer 2 (chocolate) sand from 10–20 cm was moved upward into the native layer at 0–10 cm. About 42 g of layer 3 (green) sand from 20–30 cm was deposited in the native layer (0–10 cm) and about 10 g in layer 2 (chocolate, 10–20 cm). These numbers diminish with depth (Figs 8 and 9), so that most source layers below 50 cm are represented by 1 to 8 g in the top two layers (0–20 cm), and less than one g below that. A few deposits of 1–3 g occurred below 80 cm, mostly of sand moved downward.

Fig 8 also shows what proportion of this sand was moved up and down. The proportion moved and deposited upward decreased with depth, reaching a minimum of 71% in purple layer 9 (purple, 80 to 90 cm), and increasing again toward the nest bottom. Necessarily, the amount moved and deposited downward increased from 2% in layer 2 (chocolate, 10 to 20 cm) to a maximum of 29% in layer 9 (purple, 80 to 90 cm). Of course, the scope for downward movement decreased as the bottom of the nest was approached, so that no layer 12 sand (divot) was discovered in layer 13 (red clay, 160–210 cm).

**Fig 10:**
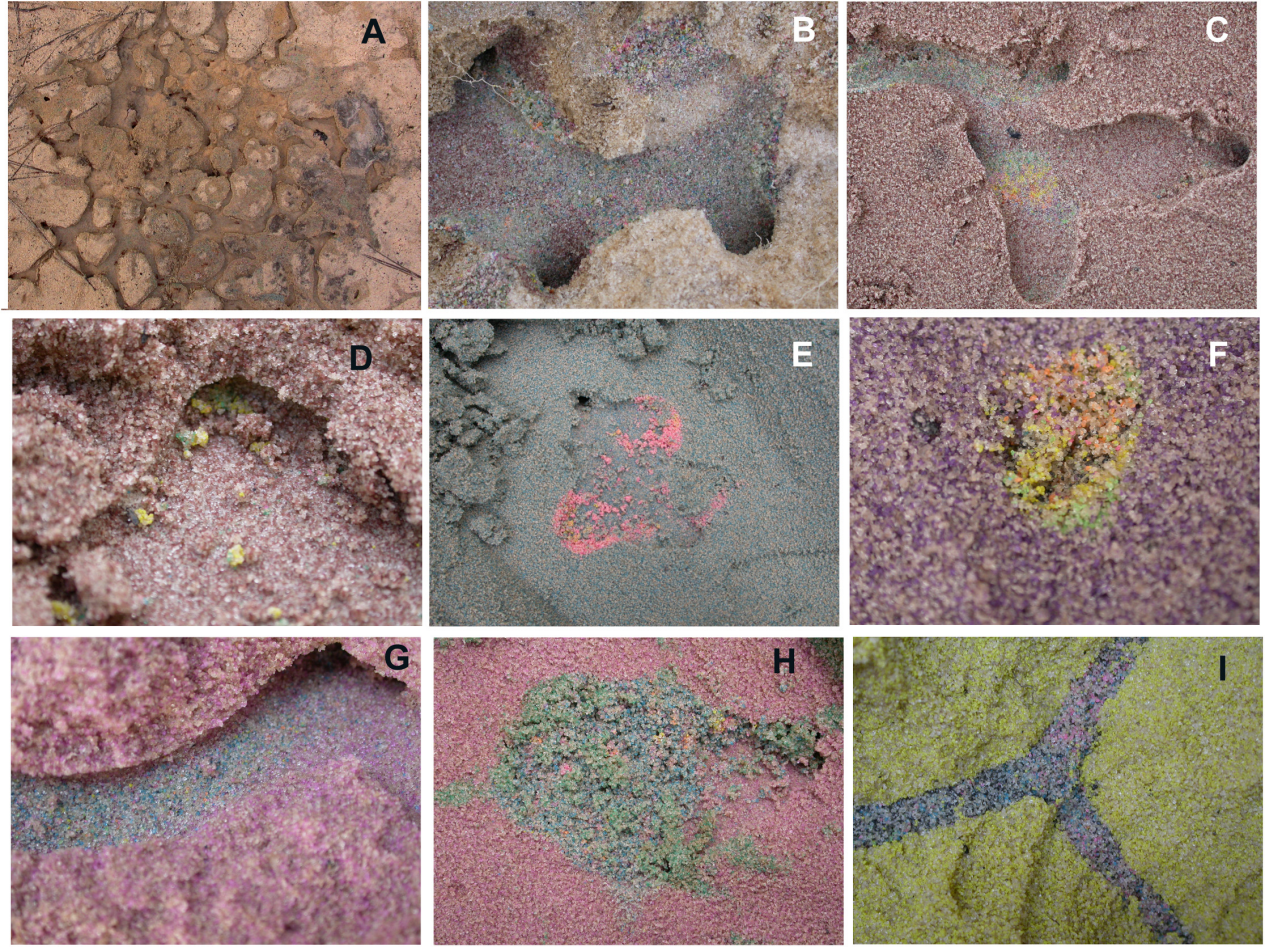
a-i. Examples of non-host sand deposition below ground. A. Chambers immediately under the screen cage ~1–2 cm. Backfills are visible at higher magnification. B, C, G show deposition smoothed and incorporated in to chamber and shaft walls and floors. F, I are shafts backfilled with colored non-host sand. D, E, H show pellets of colored sand deposited in chambers.

Examples of non-host sand deposits are shown in Fig 10A–10I. Deposition took several forms: (1) back-filling existing chambers, particularly common in the uppermost regions of the nest (Fig 10A), but also in deeper regions (Fig 10E and 10H); (2) lining the floors and walls of chambers (Fig 10B and 10C); (3) lining the floors and walls of shafts (Fig 10G); (4) filling shafts (Fig 10F and 10I) and (5) dropping formed pellets in chambers (Fig 10D and 10H). In all of these cases, several colors were mixed together, suggesting that deposition events were repeated many times with sand pellets from multiple depths. For example, 6 to 8 colors of sand are present in Fig 10B, 10C and 10H. Even some of the yellow pellets in Fig 10D contain grains of green and orange. The pink backfill in Fig 10E is unusual in consisting mostly of pink pellets.

Overall, 42% of the weight of below-ground deposits was in backfills, 40% in chamber floors, 1% in filled shafts and 16% was not classified. Of the 1009 cm^2^ area of the uppermost chamber in the native layer (0–3 cm depth), about 20% (210 cm^2^) was backfilled. Altogether, of the 209 g of deposition in layer 1 (native, 0–10 cm), 109 g was backfill. Backfilling also occurred in deeper regions but constituted a smaller percent of the chamber area. Of the 6 instances of backfill in deeper layers, about 24 g of the total 137 g deposited was in backfills. Nevertheless, backfilling clearly accounts for more deposition than chamber lining or filling of shafts.

### Mixing colors

**Fig 11:**
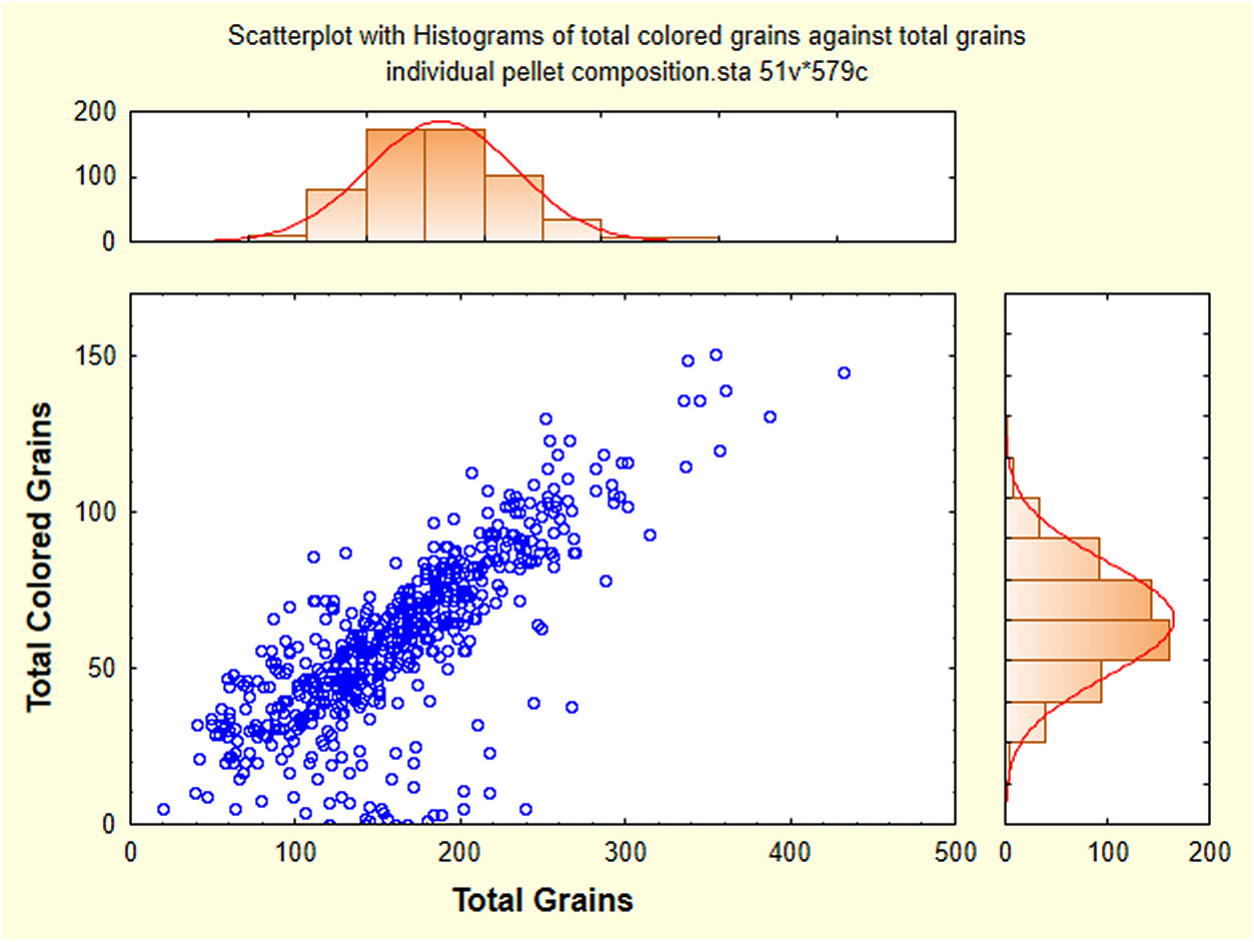
The number of colored grains in whole sand pellets in relationship to the total number of grains. The slope indicates that about a third of the grains were colored. The histograms show the frequency distribution of total grains and colored grains.

**Fig 12:**
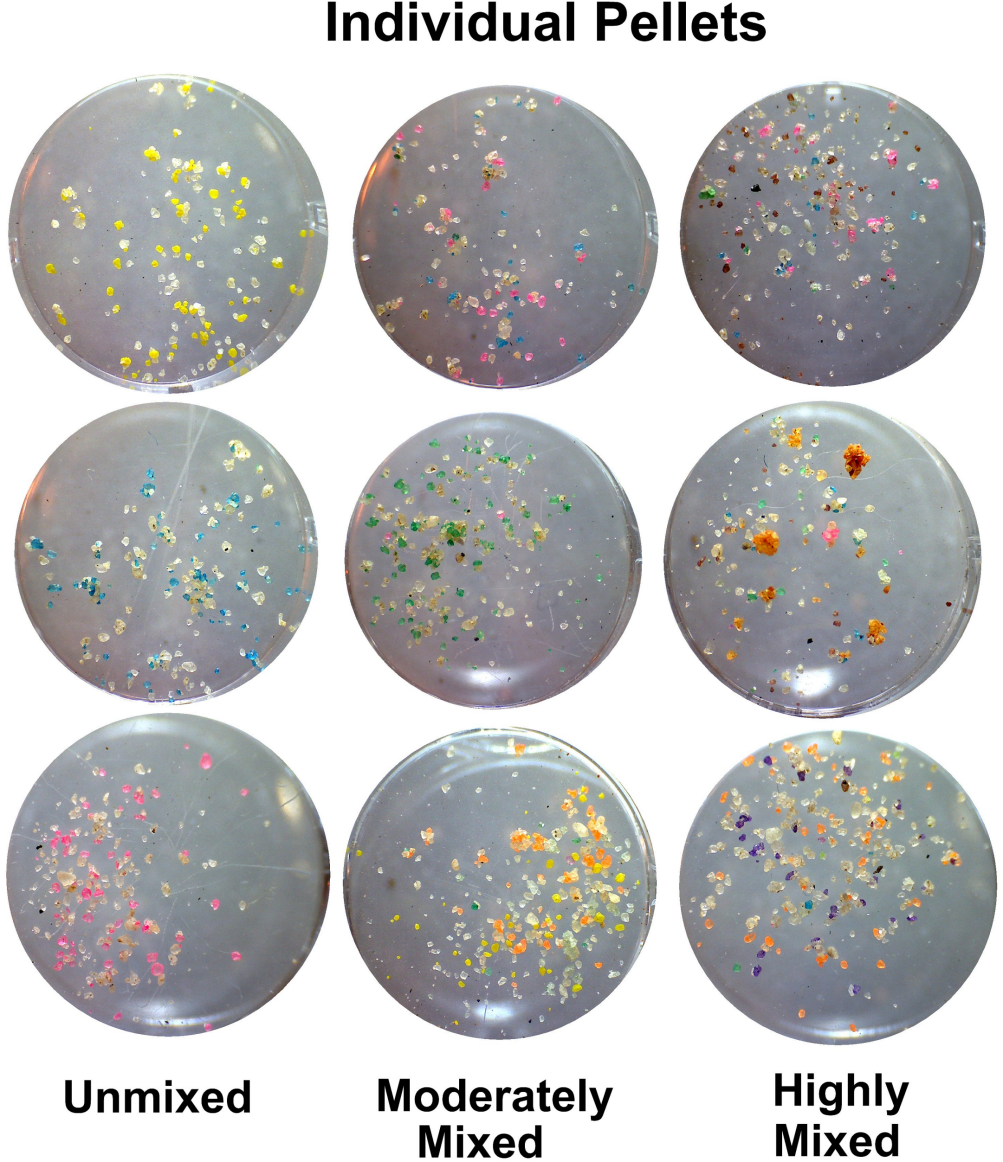
Examples of individual pellets that were unmixed in color, moderately mixed and highly mixed. Highly mixed pellets sometimes contained 10 of the 12 colors. Each well contains only a single pellet.

To address the question of when the mixing of colors occurs, 20 to 48 individual pellets per day were collected whole as workers deposited them at the surface between Oct. 21 and Nov. 13. These were photographed individually and analyzed as for the daily samples. Fig 11 shows that pellets contained from about 50 to 400 grains of sand, with a mean of 162 total grains of which 60 were colored. On average, pellets were about 35% colored grains. By the time pellets reached the surface, a large fraction of them contained more than one color of sand. *Yet when the pellets were first formed at the growing margin of a chamber or shaft*, *the vast majority had to consist of a single color*. This suggests that pellets were often deposited and reformed on the way to the surface. Examples of unmixed, moderately mixed and highly mixed pellets are shown in Fig 12.

Overall, the number of colors in a pellet ranged from 0 to 10 (a single pellet had 12), with an overall mean of 4.36 (s.d. = 2.07, n = 579). The mean number of colors varied from about 3.1 to 5.7 between Oct. 21 and Nov. 13, 2011 (one-way ANOVA, p< 0.001). This variation approximately reflected the relative sand removal rates seen in Fig 4-—lower when sand came predominately from a few layers, and higher when activity was spread more evenly among all the layers.

**Fig 13:**
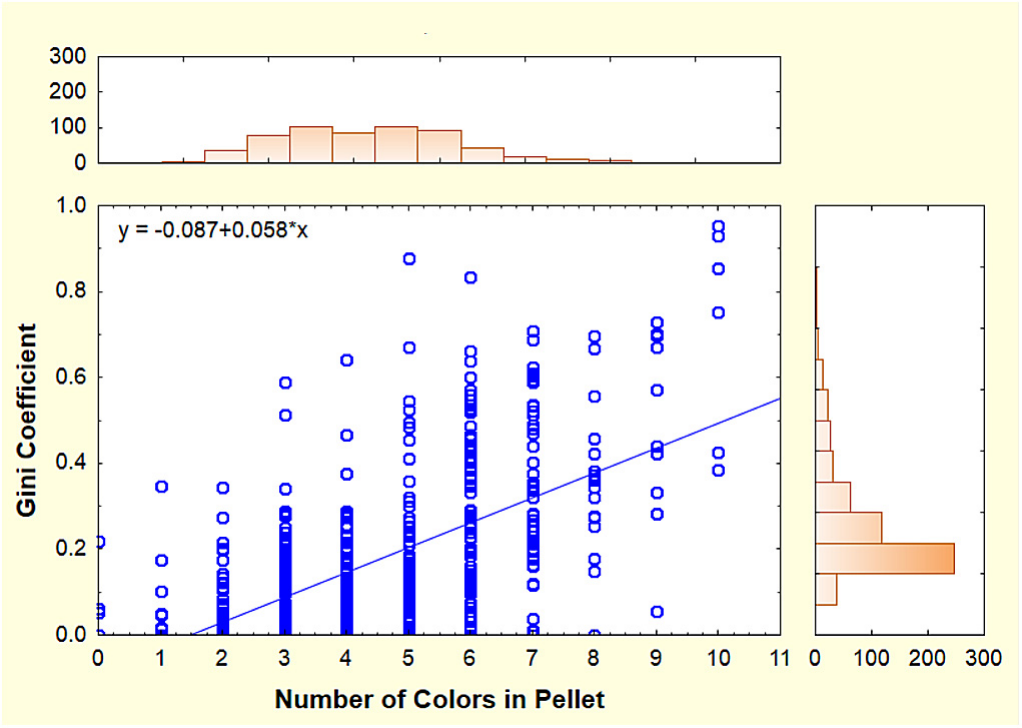
The Gini coefficient of individual pellets estimates the evenness of colored grain representation, with a coefficient of zero indicating that only a single color made up the pellet, while a coefficient of 1.0 indicates a pellet in which all colors present were equally represented. The Gini coefficient increased by about 0.6 for every additional color in the pellet. Histograms show the frequency distribution of number of colors (top histogram) and Gini coefficients (right histogram).

**Fig 14:**
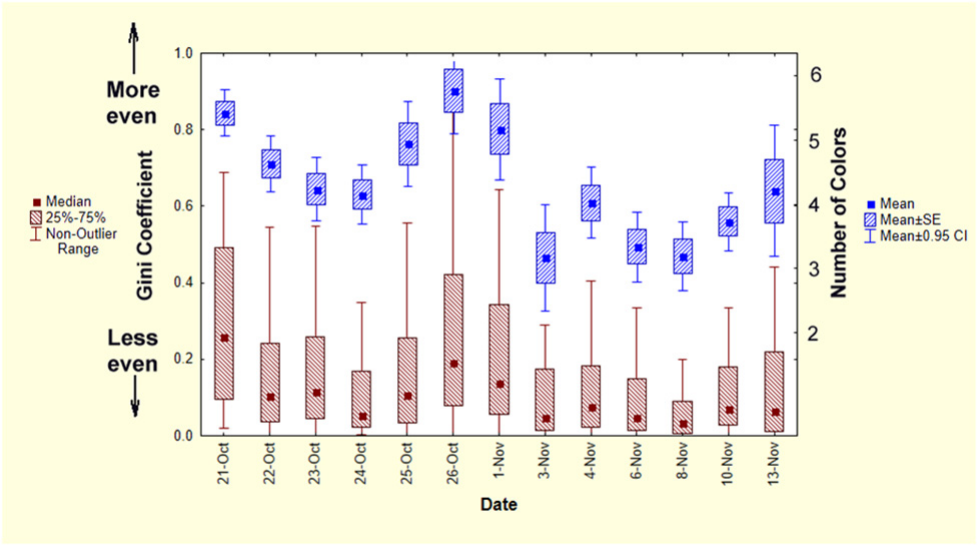
The Gini coefficient of pellets changed with date, showing that mixing was not constant. Mean number of colors (blue, right scale) and the evenness of color representation shown as the median Gini coefficient (brown, left scale). With more colors, colors were more evenly represented.

The number of colors in a pellet still left open the question of how evenly or unevenly the colors were represented. Evenness was estimated by computing Gini coefficients for colored grains in all pellets from Oct. 21 to Nov. 13, 2011 (native grains were excluded). A Gini coefficient of 0 indicated all colored grains were of a single color, while at the other extreme, a coefficient of 1.0 indicated that multiple colors were present, each represented by the same number of grains, i.e. complete evenness. Fig 13 shows that as the number of colors in a pellet increased, their evenness increased as well, i.e. the Gini coefficient increased by about 0.06 for every additional color. Nevertheless, the mode of the Gini coefficient was between 0.1 and 0.2 (Fig 13, right histogram), indicating that in most pellets, colors were unevenly represented. In spite of this, even with up to 6 colors in a pellet, some pellets had very low Gini coefficients, probably because most colors were represented by very few grains, or even a single grain. The Gini coefficients and the mean number of colors both varied significantly and more or less in parallel by date (Fig 14; Kruskal-Wallis median test; Chi-square = 47.1, d.f. = 12; p< 0.0001) and activity in the colored layers (Fig 4).

Together, the deposits of non-host sand and the analysis of individual sand pellets suggest that a substantial fraction of sand excavated below is not carried directly to the surface, but is deposited and formed into pellets multiple times *en route*, leading to the observed mixture of colors in the pellets. It is also possible that pellets acquire grains by accidental contact with tunnel or chamber walls, or contamination from grains stuck to the worker's mouthparts. However, Fig 11 suggests that such "accidental" acquisition is relatively rare. While intact pellets were occasionally seen below-ground in chambers (Fig 10D, 10E and 10H), much of the dropped sand is smoothed into the walls and floors of chambers and shafts (Fig 10B, 10C and 10G). This suggests that the subterranean architecture of the nest is dynamic and subject to continuous remodeling. This is particularly apparent in the uppermost chambers in which backfilling and re-excavation seem to occur frequently.

### Sand caching: Short-term movement and deposition

**Fig 15:**
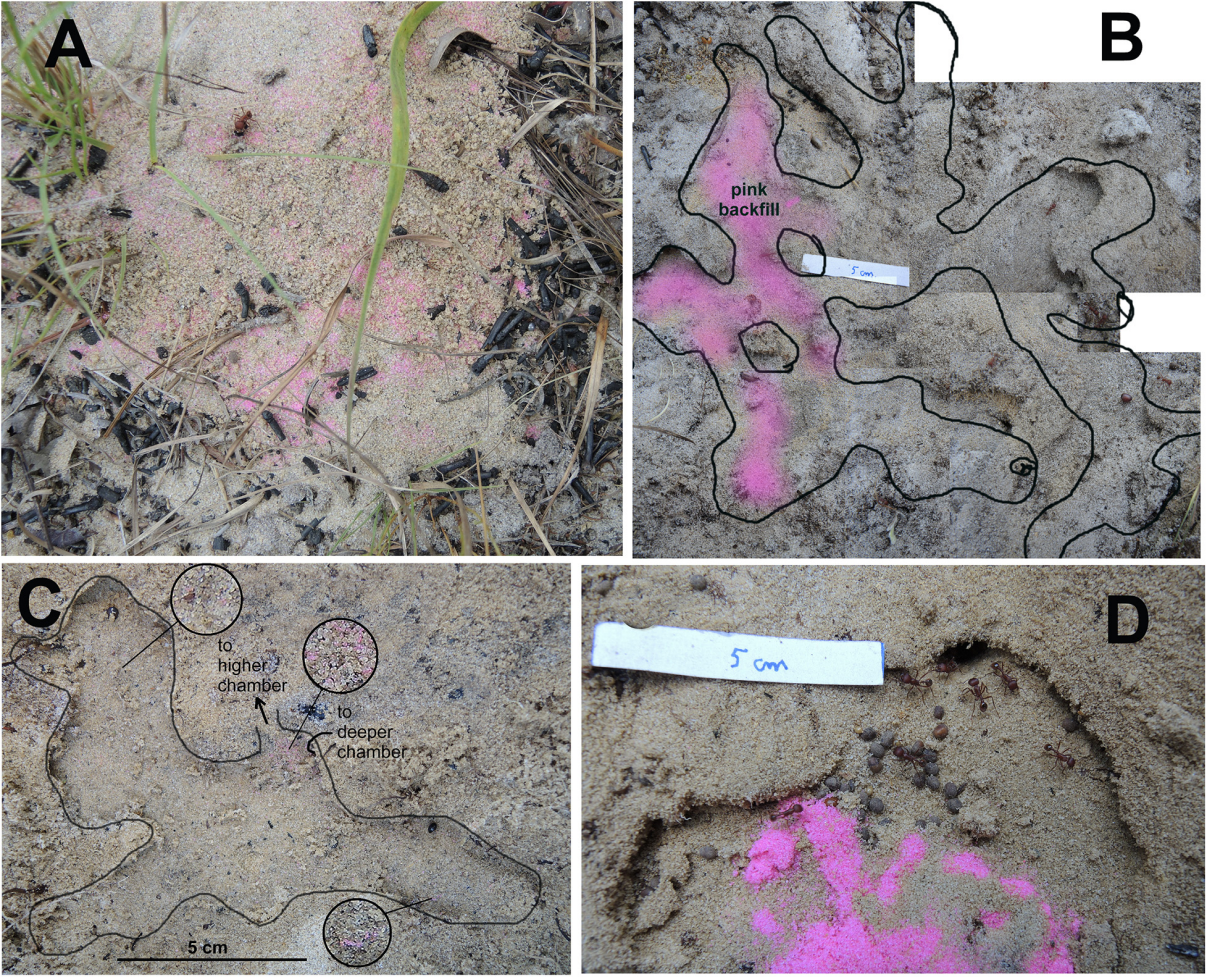
Sample images from the pink sand experiment. A. Pink sand pellets and grains on the surface disc. B. Heavy deposition and backfill of pink sand in a chamber at 2 cm depth, just below the surface. Chamber outlines shown by the black line. C. Scattered grains and light deposition in a deeper chamber. Chamber outline shown by black line. Circles are magnified vignettes from the indicated location, showing scattered grains of pink sand or light deposition. D. Chamber in which the pink sand was injected.

The deposition observed in the layer cake dig was created between October 2011 and May 2012, and it is not possible to determine when or how rapidly it was created. Is deposition a constant and continuous part of nest excavation, or does it occur in particular phases of this process? To address this question, pink fluorescent sand was placed into a 30 to 50 cm deep nest chamber from a lateral pit. One to three days later, the nest disc was checked for pink sand pellets, and the nest excavated chamber by chamber to check for deposits of pink sand or pellets of pink sand. Pink sand was visible in the nest disc in 6 of the 7 nests (Fig 15A), and excavation showed that of 42 chambers in the 7 nests, 27 contained scattered to many grains of pink sand (e.g. Fig 15B and 15C), one was backfilled with pink sand, one contained intact pellets and 8 had no evidence of pink sand. The backfilled chamber was an uppermost one, and pink-free chambers were mostly of intermediate depths. Fig 15D shows the chamber into which the pink sand was emplaced. This experiment showed that "contamination" of nest levels by sediment excavated from below is an integral part of excavation in which some fraction of the transported sand is dropped and picked up more than once before reaching the surface.

The terminal layer cake nest excavation represents a snapshot in time, that is, the final condition six months after installation of the colony. This pink sand implantation experiment showed that deposition and reformation is an immediate and continuous process, so that had more time elapsed before evaluating the layer cake nest, the non-host deposits, while still present, would have been different in composition and location.

A possible criticism of this experiment is that this form of transport might be a response to damage rather than the normal process of excavation. However, intact, deposited pellets were also observed in the layer cake experiment (e.g. Fig 10D, 10E, 10F and 10H), suggesting that such deposition is not an artifact of nest damage.

### Seed caching

**Fig 16:**
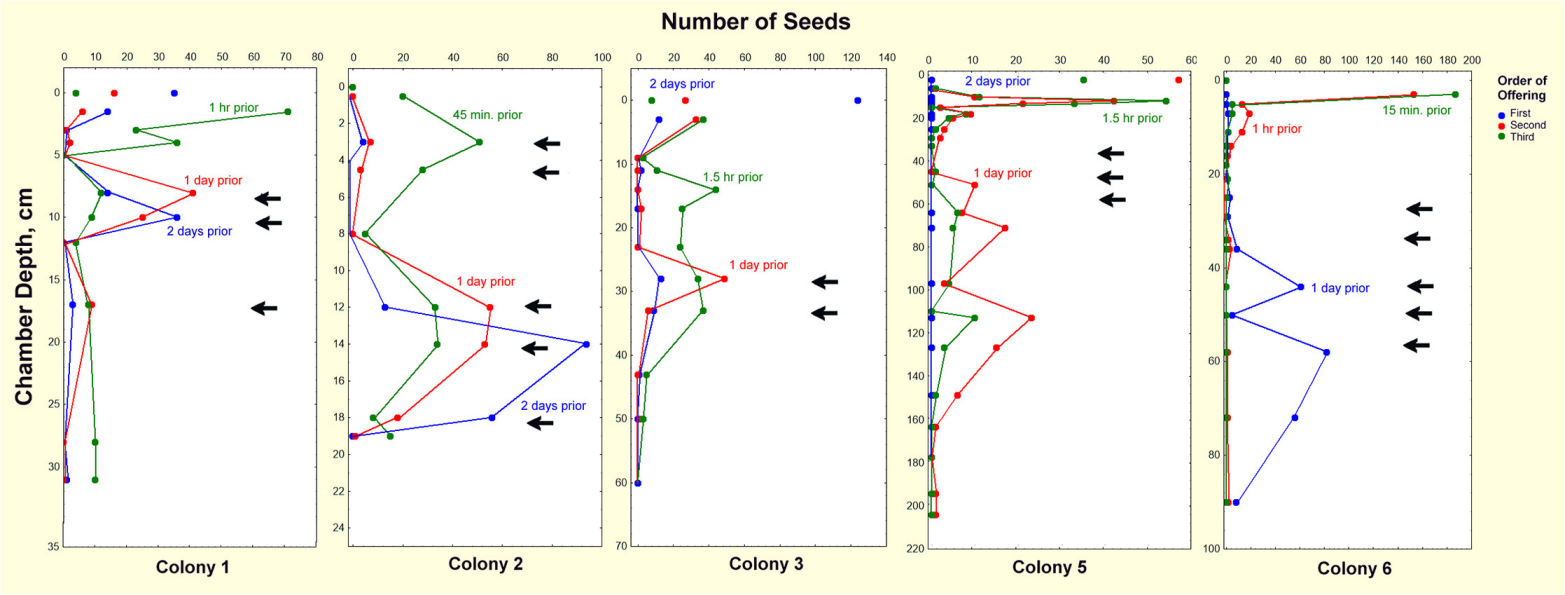
Seed caching during downward transport. Colonies were offered dyed seeds 1 and 2 days before, and shortly before excavating the colony. Arrows indicate seed chambers. Colonies 2 and 6 were unusual in having very shallow seed chambers.

When the foragers had moved the third offering of seed into the nest, all foragers were removed with a vacuum so that no further seeds entered the nest, and nest excavation commenced. The most recently offered seeds (15 min. to 1.5 hr previously) were most abundant in the uppermost chambers (Fig 16). Nevertheless, many of these seeds had been carried deeper into the nest within less than an hour. Seeds collected one day previously were mostly deeper in the nest, and those collected 2 days previously were deeper still. These patterns suggest that seed transport is a partitioned task–—foragers drop seeds in the uppermost chambers (foragers are never found below about 12 cm), from which other workers within the nest shuttle them downward in stages. This is particularly clear in Colonies 1, 2 and 3 in which the most recent seeds appear to be moved down in a wave that has not yet penetrated to the depths of seeds acquired one to two days previously. In Colony 6, seeds collected 15 minutes previously were limited to the uppermost chamber, while seeds collected an hour earlier had been partly moved to three deeper chambers.

### Overlap of sand transporters and seed foragers

While only 16% (s.d 0.10) of foragers from the initial sample survived until day 18, forager replacement exceeded loss, and each colony’s forager population grew an average of 1.15 (s.d 0.14) times its original size. The sand-transporter population was up to two times the size of the forager population in each colony (mean = 1.50, s.d 0.89). Double marking revealed that 38% (s.d 5.7%) of sand transporters also belonged to the forager population on day one. These sand-transporters, marked as foragers, died at approximately the same rate as the general forager population, and only 18% (s.d 5.0) survived after 18 days. However, 40% (s.d 0.10%) of the original sand worker population was captured foraging on day 18. Therefore, survival in the initial sand-transporter population was *at least* 2.5 times higher than survival in the forager-only population, and sand transporters gradually continued to become foragers over 18 days. Sand transporters marked on day one, accounted for a maximum of 89% of new foragers present on day 18, suggesting that the tendency of a sand transporter to forage, and her risk of mortality increased with time. Sand-transport and foraging seem to be overlapping tasks but the progression seems to be from sand-transporting to foraging as the workers age. The world of the forager is surely of a larger dimension than that of a sand-transporter.

## Discussion

Whereas this study does not solve the mystery of how groups of workers, without a blueprint and without a leader create a subterranean nest in the dark, it does provide a description of some of the rates and patterns of the processes involved. Nest excavation occurs in association with nest relocation, so that the gradual excavation of the nest and the relocation of the colony into it by means of a two-way trail go hand in hand [1, 35]. In 4 to 6 days, the process is complete, and the colony resides in a new nest, having recreated a similar nest and a similar vertical distribution of its members within. In a natural relocation, a subgroup of foragers initiates the new nest, and other workers gradually elect to join this group. The younger members of the forager class tend to carry out the bulk of the sand-transport, a situation we tried to simulate by incrementally adding workers to the nest excavation, but the architectural details of the resulting nest deviated from the natural. We had no way of selecting the "right" workers to add, nor was a two-way trail present. Nevertheless, the vertical distribution of chamber area, as well as the total area and maximum depth were in line with a natural nest of about 10,000 workers.

The layer cake experiment demonstrates the orderliness of the nest excavation, revealing as it does the location and rate of nest deepening and chamber enlargement, and allows quantification of the volume of sand excavated in relation to depth and elapsed time. The colors in the daily take of sand showed that nest deepening and chamber enlargement occur simultaneously in a consistent proportion, so that the nest always has its species-typical proportions, no matter what size or stage [8]. Although the nest is essentially complete in two weeks, it is almost doubled in volume in the early spring following a period of inactivity during the winter, showing that nest volume is not a simple multiple of the nest population.

Evidence was indirect but strong that workers digging at the margins of growing chambers or deepening shafts do not themselves carry the sand pellets they create all the way to the surface. Rather, upward sand transport is partitioned and occurs through a sequential chain of transport, deposition, and re-transport, until the pellet is either dumped on the ground surface, or is incorporated into the wall or floor of a chamber or shaft. Even from here, the sand can be mobilized once again, as evidenced by the frequency of pellets with multiple colors of sand. The evidence from the pink sand experiment was direct and showed that this process is immediate and continuous.

Such a multi-step process is in accord with the laboratory findings of Pielstrom and Roces [16] who found that workers excavating pellets of soil at the chamber wall deposit these pellets only a short distance away, each excavator having a preferred place of deposit, separate from others. From there, pellets are moved toward the nest exit first by short-distance transporters and then by long-distance transporters who eventually deposit the pellets outside the nest. Total transport may involve as many as 12 different workers belonging to the three labor groups. Excavators also stridulate while cutting pellets, and this stridulation attracts other workers to the site and increases the likelihood that they will dig too [47]. The efficiencies created by this arrangement are that excavators work very locally and are not faced with the problem of finding their way back to the work site, which in both *Atta vollenweideri* and *P*. *badius*, can be several meters from the final deposition site. Moreover, in *A*. *vollenweideri*, the deposits of fresh pellets act as stigmergic cues that organize collective nest excavation by attracting both short-distance transporters and additional excavators (in stigmergy, changes in the condition of the shared task indirectly coordinate the behavior of workers without direct communication). Stigmergy may be important in the creation of specific nest architectures in this and other ant species, but how it might do this is currently unknown.

A probable difference between *P*. *badius* and *A*. *vollenweideri* is that the sand pellets produced by the former are fragile, while the clay pellets of the latter are not. It is possible that it is this fragility, combined with the vagaries of sequential transport and deposition that leads to the incorporation of soil from below into the walls and floors higher in the nest. Whether this occurs in *A*. *vollenweideri* is not known, but would appear less probable in view of the lower fragility of its clay pellets. This implies that the nature of the soil in which a nest is being excavated may affect the rate of subsurface incorporation, i.e. that this rate is not simply a function of ant behavior.

Vegetation-cutting and transport in Attine ants are partitioned in a manner similar to nest excavation, with leaf pieces acting as stigmergic cues to transporters, and sequential transport more pronounced in longer trails [20, 21]. Similarly, in the seed harvesting ant, *Messor barbarus*, seeds not transported directly from their source to the nest are either transferred from small workers to large worker in a ‘bucket brigade’, or deposited in temporary caches along established foraging routes [18, 19, 22]. Partitioning of transport tasks by direct transfer (relay) and indirect transfer (caching) have been observed in numerous ant and termite species [20, 48–50] and may allow experienced workers to deplete a food source or excavation zone more rapidly without spending time queuing in congested nest space [49, 51]. Likewise, spatial localization of labor may promote colony health by minimizing contact between exterior workers and interior workers, reducing the transmission of pathogens acquired outside the nest [25, 27, 52, 53].

Just as sand is transported upward in stages, seeds are transported downward in sequential stages. *P*. *badius* foragers drop their seeds in upper chambers and never travel deeper than 10 to 15 cm in the nest. Though within the nest, foragers are limited to this space, they represent less than half of the ants found in the uppermost 20 cm of the nest [44]. This suggests that the downward movement of seeds is accomplished by a different set of workers, possibly the same workers that relay sand upward. Within hours to a day, most of the seeds deposited by foragers come to reside in seed chambers more than 30 cm below the surface, suggesting that seeds act as stigmergic cues for seed-bearing transporters to drop their seeds in much the same way that necrophoric workers drop their burdens upon encountering piles of dead workers [54]. Thus, individual pellets or seeds may stimulate carrying, but piles of each may stimulate deposition, with considerable leakage along the way.

The movement of soil by ants is of great interest to both geology and archeology because it has the capacity for scrambling the age and deposition signals of soils. The below-ground deposition rate of sand from deeper levels confirms the general results of Rink et al. [42] and quantifies deposition by source and level for a total of 2.5% of the excavated volume of sand. Thus, OSL dates should be most "compromised" just below the surface where backfilling of chambers is common, and decrease to a minimum at about 50 to 70 cm. The expected effects of below-ground deposition on OSL dates is thus somewhat regular, at least for a single nest result as we present here. More than 240 g of deeper sand was incorporated in the dark into the top 30 cm, or 0.4% of this region. This addition was not homogeneous but distributed as scattered hotspots. An OSL sample that included such a hotspot would provide a very erroneous age overestimate if based on the mean luminescence (central age model OSL age [30]). Over many generations of nests, the upper layers of sediment would come increasingly to contain deeper, older sand, perhaps about 0.4% per generation in the top 30 cm if nest sites were exactly congruent, but such estimates would also need to take into account the downward movement of the upper layers through burial as a result of biomantling above. From 60 to 90 cm, a significant percentage of sand is moved downward, an observation also made by Halfen and Hasiotis [55] in laboratory colonies of *P*. *californicus*.

## Supporting Information

S1 Data Mean grain counts from daily sand samples. Daily amount of sand excavated, and the level origin were calculated from these data.
(XLS)

S2 Data Count of the sand colors in individual pellets. (XLS)
